# Supplementary material for: Role and mechanism of NCAPD3 in promoting malignant behaviors in gastric cancer
Source: Front Pharmacol. 2024 Apr 22;15:1341039. doi: 10.3389/fphar.2024.1341039 (PMC11070777; doi:10.3389/fphar.2024.1341039)
Supplement: Supplementary file 11 [file DataSheet2.ZIP › GSEA/immunologic/my_analysis.Gsea.1599462003817/GSE1460_INTRATHYMIC_T_PROGENITOR_VS_CD4_THYMOCYTE_DN.html]

Details for gene set GSE1460\_INTRATHYMIC\_T\_PROGENITOR\_VS\_CD4\_THYMOCYTE\_DN[GSEA]

|  || Dataset | filtered\_dataset.sample\_info.cls#WT\_versus\_NCAPD3\_MUT |
| Phenotype | sample\_info.cls#WT\_versus\_NCAPD3\_MUT |
| Upregulated in class | NCAPD3\_MUT |
| GeneSet | GSE1460\_INTRATHYMIC\_T\_PROGENITOR\_VS\_CD4\_THYMOCYTE\_DN |
| Enrichment Score (ES) | -0.6699139 |
| Normalized Enrichment Score (NES) | -2.6768706 |
| Nominal p-value | 0.0 |
| FDR q-value | 0.0035580704 |
| FWER p-Value | 0.009 |
Table: GSEA Results Summary

  

Fig 1: Enrichment plot: GSE1460\_INTRATHYMIC\_T\_PROGENITOR\_VS\_CD4\_THYMOCYTE\_DN      
 Profile of the Running ES Score & Positions of GeneSet Members on the Rank Ordered List

  

| SYMBOL | TITLE | RANK IN GENE LIST | RANK METRIC SCORE | RUNNING ES | CORE ENRICHMENT || 1 | 104 | ADARB1 | 518 | 0.463 | -0.3208 | No |
| 2 | 3106 | HLA-B | 855 | -0.258 | -0.5335 | No |
| 3 | 3716 | JAK1 | 939 | -0.363 | -0.5536 | No |
| 4 | 10019 | SH2B3 | 1044 | -0.440 | -0.5804 | No |
| 5 | 27250 | PDCD4 | 1170 | -0.537 | -0.6116 | Yes |
| 6 | 5154 | PDGFA | 1191 | -0.566 | -0.5646 | Yes |
| 7 | 7128 | TNFAIP3 | 1198 | -0.573 | -0.5067 | Yes |
| 8 | 330 | BIRC3 | 1224 | -0.600 | -0.4596 | Yes |
| 9 | 2887 | GRB10 | 1259 | -0.646 | -0.4138 | Yes |
| 10 | 7414 | VCL | 1308 | -0.712 | -0.3709 | Yes |
| 11 | 4739 | NEDD9 | 1322 | -0.741 | -0.2999 | Yes |
| 12 | 113791 | PIK3IP1 | 1345 | -0.787 | -0.2302 | Yes |
| 13 | 27076 | LYPD3 | 1348 | -0.790 | -0.1459 | Yes |
| 14 | 54463 | FAM134B | 1356 | -0.815 | -0.0624 | Yes |
| 15 | 6711 | SPTBN1 | 1381 | -0.925 | 0.0208 | Yes |
Table: GSEA details [plain text format]

  

Fig 2: GSE1460\_INTRATHYMIC\_T\_PROGENITOR\_VS\_CD4\_THYMOCYTE\_DN      
 Blue-Pink O' Gram in the Space of the Analyzed GeneSet

  

Fig 3: GSE1460\_INTRATHYMIC\_T\_PROGENITOR\_VS\_CD4\_THYMOCYTE\_DN: Random ES distribution      
 Gene set null distribution of ES for **GSE1460\_INTRATHYMIC\_T\_PROGENITOR\_VS\_CD4\_THYMOCYTE\_DN**
